# Cost and quality of life analysis of HIV self-testing and facility-based HIV testing and counselling in Blantyre, Malawi

**DOI:** 10.1186/s12916-016-0577-7

**Published:** 2016-02-19

**Authors:** Hendramoorthy Maheswaran, Stavros Petrou, Peter MacPherson, Augustine T. Choko, Felistas Kumwenda, David G. Lalloo, Aileen Clarke, Elizabeth L. Corbett

**Affiliations:** Division of Health Sciences, University of Warwick Medical School, Gibbet Hill Campus, Coventry, CV4 7AL UK; Malawi-Liverpool-Wellcome Trust Clinical Research Programme, Blantyre, Malawi; Department of Public Health and Policy, University of Liverpool, Liverpool, Merseyside L69 3BX UK; Department of Clinical Sciences, Liverpool School of Tropical Medicine, Pembroke Pl, Liverpool, L3 5QA UK; London School of Hygiene and Tropical Medicine, London, UK

**Keywords:** HIV, HIV testing and counselling, HIV self-testing, Costs, Health-related quality of life, EQ-5D

## Abstract

**Background:**

HIV self-testing (HIVST) has been found to be highly effective, but no cost analysis has been undertaken to guide the design of affordable and scalable implementation strategies.

**Methods:**

Consecutive HIV self-testers and facility-based testers were recruited from participants in a community cluster-randomised trial (ISRCTN02004005) investigating the impact of offering HIVST in addition to facility-based HIV testing and counselling (HTC). Primary costing studies were undertaken of the HIVST service and of health facilities providing HTC to the trial population. Costs were adjusted to 2014 US$ and INT$. Recruited participants were asked about direct non-medical and indirect costs associated with accessing either modality of HIV testing, and additionally their health-related quality of life was measured using the EuroQol EQ-5D.

**Results:**

A total of 1,241 participants underwent either HIVST (n = 775) or facility-based HTC (n = 446). The mean societal cost per participant tested through HIVST (US$9.23; 95 % CI: US$9.14-US$9.32) was lower than through facility-based HTC (US$11.84; 95 % CI: US$10.81-12.86). Although the mean health provider cost per participant tested through HIVST (US$8.78) was comparable to facility-based HTC (range: US$7.53-US$10.57), the associated mean direct non-medical and indirect cost was lower (US$2.93; 95 % CI: US$1.90-US$3.96). The mean health provider cost per HIV positive participant identified through HIVST was higher (US$97.50) than for health facilities (range: US$25.18-US$76.14), as was the mean cost per HIV positive individual assessed for anti-retroviral treatment (ART) eligibility and the mean cost per HIV positive individual initiated onto ART. In comparison to the facility-testing group, the adjusted mean EQ-5D utility score was 0.046 (95 % CI: 0.022-0.070) higher in the HIVST group.

**Conclusions:**

HIVST reduces the economic burden on clients, but is a costlier strategy for the health provider aiming to identify HIV positive individuals for treatment. The provider cost of HIVST could be substantially lower under less restrictive distribution models, or if costs of oral fluid HIV test kits become comparable to finger-prick kits used in health facilities.

**Electronic supplementary material:**

The online version of this article (doi:10.1186/s12916-016-0577-7) contains supplementary material, which is available to authorized users.

## Background

Awareness of HIV status is key to ensuring timely access to effective HIV treatment and prevention [1, 2]. Sub-Saharan Africa accounts for three quarters of all new infections and HIV-related deaths [3], and despite massive increases in funding for HIV testing services, only one half of Africans know their HIV status [3, 4]. New targets, set by UNAIDS (“90-90-90”), and agreed to by most African countries including Malawi, are for 90 % of Africans living with HIV to know their status by 2020, with 90 % of these retained on antiretroviral therapy (ART), and 90 % of those on ART having undetectable viral loads [5]. Uptake, however, remains low in hard-to-reach populations, including men and adolescents, and amongst those who do have regular contact with broader healthcare services, including pregnant women and those with tuberculosis (TB) disease [3].

HIV testing and counselling (HTC) continues to be undertaken predominantly in health facilities [4], despite strong evidence to suggest clients prefer to test in the community [6, 7]. Community-based HTC, including home-based and mobile services, reach HIV infected individuals earlier in their disease progression [8], potentially improving health outcomes and reducing healthcare costs of care provision [9]. Community-based HTC may be essential to reach the 90-90-90 targets [10], but costs tend to be higher than for facility-based HTC services, with lower uptake of post-test HIV care services unless facilitated linkage interventions are provided alongside [11, 12].

HIV self-testing (HIVST) has been found to be highly acceptable, safe and effective at achieving high coverage rates in communities, including amongst hard-to-reach populations of men and adolescents [13–15]. However, no primary cost analyses have been undertaken of HIV self-testing services in sub-Saharan Africa to inform policy, hindering efforts to design scalable implementation strategies. We undertook a costing study to investigate the costs to both healthcare providers and users accessing either HIVST or facility-based HTC. We additionally describe the health-related quality of life of users of these services. We collected individual-level economic data from users of both services, and undertook primary costing studies of the two approaches, within the context of a large cluster-randomised study investigating the impact of offering HIVST in addition to facility-based HTC in Blantyre, Malawi.

## Methods

### Study design and participants

The study recruited individuals who were participants in a cluster-randomised trial (ISRCTN02004005) investigating the impact of offering HIVST in addition to standard facility-based HTC [16]. We estimated the economic costs associated with HIVST and facility-based HTC, and additionally the health-related quality of life (HRQoL) of participants accessing either modality.

The study area included three high-density urban suburbs of Blantyre, Malawi [15] with an adult population of approximately 34,000 residents and adult HIV prevalence of 18 % [13]. Twenty-eight clusters of approximately 1,200 adults were randomised to either HIVST or standard-of-care (control). In all clusters, participants could access HTC at the health facilities by self-presenting or after referral by medical personnel. In the 14 intervention HIVST clusters, resident volunteer counsellors promoted HIVST and provided pre- and post-test counselling, as well as directions on how to use the self-test kits. Participants could self-test in the privacy of their own homes.

Routine and confirmatory HIV testing and care services were available at Queen Elizabeth Central Hospital (QECH), and two primary health clinics located in the study area (Ndirande Health Centre, Chilomoni Health Centre). HIVST was provided for a two-year period, with the service introduced from February to May 2012. From the onset of intervention in seven HIVST clusters, and from January 2013 in all HIVST clusters, participants could also request home-based assessment and initiation of 14 days of HIV care, including ART if eligible [14]. Subsequent care was provided at the primary care level.

The present study recruited participants from February 2013 to April 2014. Recruitment was restricted to adult residents of the 28 clusters who had just tested for HIV, either at home (HIVST clusters) or in a facility (all 28 clusters), but had not started ART. A previously validated satellite “Map Book” was used to determine cluster of residence [17], and consequently trial arm. Participants who accessed HIVST were recruited consecutively from the Quality Assurance (QA) cohort of the main trial [15]. The QA component systematically sampled HIVST participants, with a minimum 5 % randomly selected for home-visit by one of the trial’s study nurses. Recruitment of facility-based HTC participants was undertaken consecutively at each of the three local health facilities (Queen Elizabeth Central Hospital, Ndirande Health Centre and Chilomoni Health Centre).

### Cost analysis

Economic costing of both the HIVST service and facility HTC services was undertaken to estimate direct health provider costs [18, 19]. Costs included: staff salaries; training of staff; consumables and equipment; monitoring and evaluation; and overhead costs, as detailed in Additional file 1.

HIVST community counsellors, programme managers and accounting staff were interviewed to estimate the costs of identified resources and other service delivery, excluding research costs. For facility HTC services, HTC counsellors and administrative staff at the Blantyre District Health Office and the Queen Elizabeth Central Hospital were interviewed. Trial testing registers and Ministry of Health programme output data were used to determine overall numbers of individuals tested and number of HIV positive individuals identified.

An interviewer-administered questionnaire was developed that asked all participants recruited into the study about the direct non-medical and indirect costs that they or accompanying family member or carers incurred in accessing HIV testing services. User fees were not charged for either modality of testing. Direct non-medical costs included the cost of transportation, food and drinks whilst waiting, and other costs incurred as a consequence of testing. Indirect costs included time off work multiplied by self-reported income [20]. In addition, we recorded the total time spent testing, including travel and waiting time.

We used data reported by the World Bank to adjust all costs to account for inflation and differences in purchasing power between countries [21]. All costs are reported in 2014 US Dollars and International Dollars [19].

### Health-related quality of life

Participants were recruited after they had received their HIV test result, but before starting ART. HIV results and health-related quality of life (HRQoL) were captured at the same interview as economic costs. We used a self-assessed health (SAH) measure to ask individuals to rate their general health on a five-point Likert scale, with responses coded as: very good; good; fair; poor; or very poor. This SAH measure has been found to be a strong predictor of future health outcomes in high-income settings [22], and has also been used in resource-constrained settings [23].

A translated Chichewa version of the EuroQoL EQ-5D [24] tool was used to estimate the HRQoL of all study participants. Translation followed EuroQoL guidelines [25], and was approved before use. The EQ-5D measure consists of two principal measure components, a descriptive system and a visual analogue scale (VAS) [26]. The descriptive system defines HRQoL on the day of response in terms of five dimensions: ‘mobility’, ‘self care’, ‘usual activities’, ‘pain/discomfort’ and ‘anxiety/depression’. Responses in each dimension have historically been divided into three ordinal levels, coded: (1) no problems; (2) some or moderate problems; and (3) severe or extreme problems. Responses to the three level version of the EQ-5D place respondents into one of 243 (3^5^) health states. Resultant health states can be converted to an EQ-5D utility score using a “tariff set” derived from national surveys of the general population [26]. As no Malawian EQ-5D tariff exists, the Zimbabwean EQ-5D tariff set was used [27], assuming that Malawians will value health comparably [19]. The Zimbabwean tariff results in EQ-5D utility scores ranging from 1.0 (no problems in the five dimensions) to -0.29 (severe problems in all five dimension). The VAS, similar to a thermometer, ranges from 100 (best imaginable health state) to 0 (worst imaginable health state). Participants are asked to indicate how good their health is on the day of response by drawing a line on the VAS.

### Statistical analysis

Analysis used Stata version 13.0 (Stata Corporation, College Station, TX, USA). Comparisons of categorical variables used the chi-squared test, with the student’s *t*-test used for EQ-5D utility and VAS scores. Principal component analysis was used to generate wealth quintiles combining socioeconomic variables including nine household assets, and home environment variables [28].

Direct health provider cost per individual tested, and the cost per HIV positive individual identified were estimated from total annual provider cost of HTC services divided by number of individuals tested and number testing positive, respectively. For the HIVST, the proportion testing HIV positive was based on overall parent study data [15]. Direct health provider cost per individual assessed for ART eligibility, and per ART initiation, was estimated directly for the facility HTC cohort. The HIVST cohort data did not capture participants who were assessed for/initiated on ART by the trial home initiation option: home initiation events were, therefore, estimated from the parent trial data. National ART eligibility criteria were used (CD4 count < 350 cells/μl or WHO stage 3 or 4 or breastfeeding or pregnant).

We made comparisons between the mean direct non-medical and indirect costs for HIV self-testers and facility testers, and for facility-testers who resided in control clusters and intervention clusters. As the cost data were skewed, we used non-parametric bootstrap methods, with 1000 bootstrap replications, to derive 95 % confidence intervals (CI) for mean cost differences for relevant cost categories [29].

We undertook multivariate analysis to investigate the independent effect of the mode of HIV testing and HIV test result on the total societal costs associated with HIV testing. Total societal cost summed direct health provider costs, direct non-medical costs and indirect costs. For the HIV self-testers, we estimated the direct health provider cost per individual tested at the counsellor level. This was possible because the HIVST service records the total number of individuals tested by each of the community counsellors. For facility-based HIV testers, we used the estimated direct health provider cost per individual tested for the clinic attended for testing. As all participants incurred a cost, and the cost data were skewed, we used generalized linear models (GLM) for multivariate analyses of cost data [30]. We ran model diagnostics to determine the optimal choices for the distributional family and link function for these GLM models [31].

We compared the responses to the SAH and EQ-5D measures between HIVST participants and all facility testers, and between facility-based HIV testers residing in the intervention clusters to those residing in control clusters. For the descriptive component of the EQ-5D, binary responses (no problems or some/severe problems) were used since severe or extreme problems were rarely reported. We undertook multivariate analysis to investigate the independent effect of the mode of HIV testing and HIV test result on the EQ-5D utility score. EQ-5D utility scores were non-normally distributed, negatively skewed and truncated at 1.0. We evaluated four commonly used estimators to analyse these data: ordinary least squares (OLS) regression, Tobit regression, fractional logit regression and censored least absolute deviations (CLAD) regression [32–34]. We compared the mean squared error (MSE) and mean absolute error (MAE) statistics between the observed EQ-5D utility score and the estimated scores for the whole sample and for sub-groups of the sample based on observed EQ-5D utility scores to determine the choice of estimator.

For all multivariate analyses we ran two alternative models: the first adjusted for modality of HIV testing, HIV test result, age and sex, and the second additionally adjusted for marital status, educational attainment, income and socio-economic position [35]. We accounted for clustering in all multivariate models using the cluster of residence for the participants to produce robust variance estimators.

Sensitivity analysis was carried out using the UK York A1 tariff for the EQ-5D [36], which translates health states with ‘severe’ problems in one or more of the five dimensions into lower EQ-5D utility scores than the Zimbabwean tariff [27]. For the multivariate analysis of total societal costs, we performed additional sensitivity analyses that (i) used the median wage of the sample, and (ii) the total HIV testing time, to value income losses (Additional file 1).

### Ethical considerations

Ethical approval was obtained from the College of Medicine Ethics Review Committee, University of Malawi, and the University of Warwick Biomedical Research Ethics Committee. All participants provided written (or witnessed thumbprint if illiterate) informed consent.

## Results

**Fig. 1:**
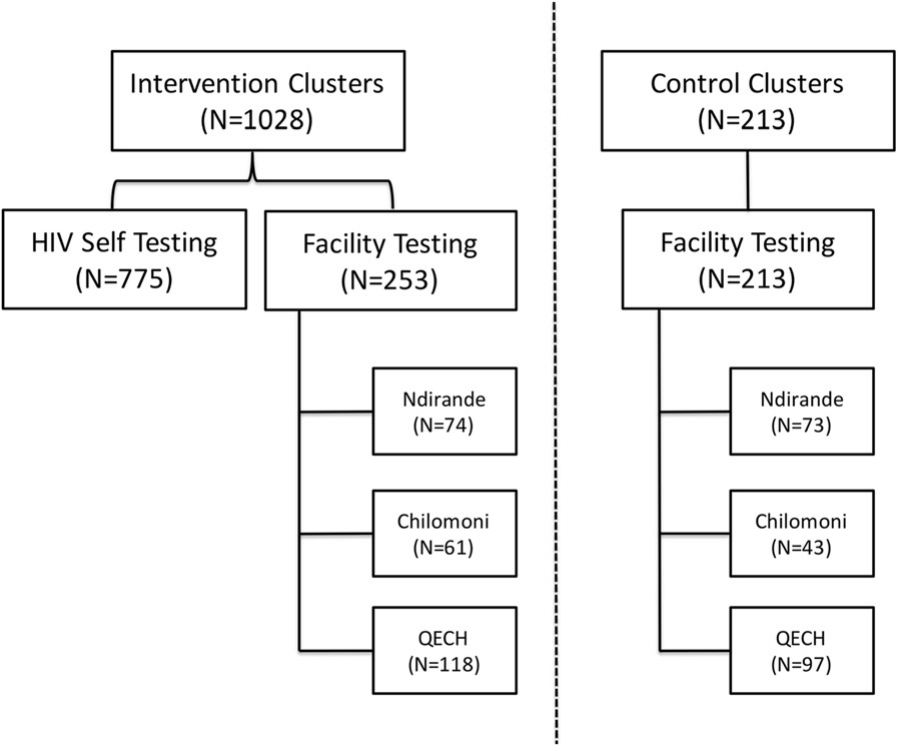
Recruitment of HIV testers by study clusters and location of HIV testing. *QECH* Queen Elizabeth Central Hospital

**Fig. 2:**
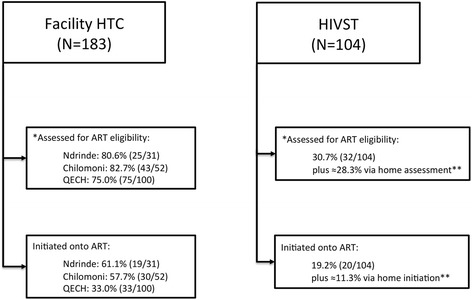
Linkage into HIV treatment after HIV testing in those eligible for assessment. *ART* Anti-retroviral therapy*, QECH* Queen Elizabeth Central Hospital. *Completed CD4 measurement or WHO stage 3 or 4, **Data from main trial. For logistical reasons, individuals assessed and initiated on ART through the home-based option were not captured in this cohort

The study recruited 1,241 participants who had either self-tested and were being assessed as part of the QA study (n = 775) or who undertook facility-based HTC (n = 466) during the study period (Fig. 1). Table 1 shows the characteristics of the participants, by residence status within the main trial and modality of HIV testing received. There were no significant differences in sex, age, marital status, educational attainment, employment or socioeconomic status between residents of intervention clusters or control clusters who accessed facility-based HTC. Figure 2 shows the estimates of linkage for those who tested HIV positive after facility-based HTC and HIVST. For facility-based HTC, 75.0 % to 82.7 % of those identified as HIV positive attended the HIV clinic for assessment for ART eligibility. For HIVST, 30.7 % of those identified as HIV positive attended the HIV clinic for assessment for ART eligibility, in addition to the estimated 28.3 % opting for home assessment of HIV care.

**Table 1:** Characteristics of HIV testers

		Intervention clusters		Control clusters	*p*-value^a^
		HIVST	Facility HTC	Facility HTC	
All		775	253	213	
Sex	Male	288 (37 . 2 %)	90 (35 . 6 %)	76 (35 . 7 %)	0 . 981
Age (years)	18-24	316 (40 . 8 %)	64 (25 . 3 %)	64 (30 . 0 %)	0 . 335
	25-39	379 (48 . 9 %)	149 (58 . 9 %)	111 (52 . 1 %)	
	40+	80 (10 . 3 %)	40 (15 . 8 %)	28 (17 . 8 %)	
Marital status	Single (never-married)	227 (29 . 3 %)	40 (15 . 8 %)	26 (12 . 2 %)	0 . 606
	Married/Cohabiting	455 (58 . 7 %)	175 (69 . 2 %)	148 (69 . 5 %)	
	Separated/Divorced	78 (10 . 1 %)	24 (9 . 5 %)	24 (11 . 3 %)	
	Widower/Widow	15 (1 . 9 %)	14 (5 . 5 %)	15 (7 . 0 %)	
Educational attainment^b^	Up to standard 8	300 (38 . 7 %)	132 (52 . 2 %)	124 (58 . 2 %)	0 . 402
	Up to form 6	442 (57 . 0 %)	113 (44 . 7 %)	82 (38 . 5 %)	
	University or training college	32 (4 . 1 %)	8 (3 . 2 %)	7 (3 . 3 %)	
Income^c^	Not working	400 (51 . 6 %)	93 (36 . 8 %)	86 (40 . 4 %)	0 . 752
	Up to 4,000 Kwacha/week	162 (20 . 9 %)	79 (31 . 2 %)	56 (36 . 3 %)	
	4,000 to 8,000 kwacha/week	108 (13 . 9 %)	42 (16 . 6 %)	34 (16 . 0 %)	
	8,000 to 12,000 kwacha/week	48 (6 . 2 %)	18 (7 . 1 %)	15 (7 . 0 %)	
	Over 12,000 kwacha/week	57 (7 . 4 %)	21 (8 . 3 %)	22 (10 . 3 %)	
Employment status	Formal employment	139 (17 . 9 %)	75 (29 . 6 %)	62 (29 . 1 %)	0 . 801
	Informal employment/Unemployed	234 (30 . 2 %)	85 (33 . 6 %)	67 (31 . 5 %)	
	School/University	159 (20 . 5 %)	18 (7 . 1 %)	15 (7 . 0 %)	
	Retired	2 (0 . 4 %)	1 (0 . 4 %)	1 (0 . 5 %)	
	Housework	238 (30 . 7 %)	72 (28 . 5 %)	68 (31 . 9 %)	
	Sick leave	2 (0 . 3 %)	2 (0 . 8 %)	0 (0 %)	
Socio-economic position^d^	Highest quintile	172 (22 . 2 %)	32 (12 . 6 %)	43 (20 . 2 %)	0 . 239
	2nd highest quintile	154 (19 . 9 %)	55 (21 . 7 %)	39 (18 . 3 %)	
	Middle quintile	148 (19 . 1 %)	58 (22 . 9 %)	42 (19 . 7 %)	
	2nd lowest quintile	145 (18 . 7 %)	55 (21 . 7 %)	48 (22 . 5 %)	
	Lowest quintile	154 (19 . 9 %)	53 (20 . 9 %)	41 (19 . 2 %)	
Had HIV testing in last year	Not tested	127 (16 . 4 %)	96 (38 . 0 %)	97 (45 . 5 %)	0 . 048
	Tested once	260 (33 . 5 %)	69 (27 . 3 %)	64 (30 . 0 %)	
	Tested >1	388 (50 . 1 %)	88 (34 . 8 %)	52 (24 . 4 %)	

^a^Comparison between facility testers in control and Intervention clusters

^b^Up to Standard 8 equivalent to completing Primary school; Up to form 6 equivalent to completing Secondary/High school

^c^426 Malawian Kwacha = US$1 in 2014

^d^Socio-economic position estimated though undertaking principal component analysis of responses to asset ownership and housing environment

Missing data for Educational attainment: 1; missing data for socio-economic position: 2

The direct health provider costs of facility-HTC and HIVST are shown in Table 2. The mean provider costs per individual tested at the three health facilities were US$7.53 (INT$20.25), US$10.57 (INT$25.18), and US$8.90 (INT$20.44) at Ndirande, Chilomoni and QECH, respectively, whilst the mean cost of providing HIVST was US$8.78 (INT$17.25). The mean provider costs per HIV positive individual identified were, however, lower at the three health facilities (range US$28.30-US$76.14) than for HIVST (US$97.50), reflecting the lower HIV prevalence among HIVST participants (9.0 %) than facility HTC participants (range 11.2 %-31.5 %). Similarly, the mean provider costs per HIV positive individual assessed for ART eligibility (facility range US$37.73 to US$92.38) and initiated on ART (facility range US$85.75 to US$132.42) were also lower at the three health facilities than for HIVST (range US$165.14-US$233.90 for eligibility assessment and US$319.67 for ART initiation).

**Table 2:** Annual direct health provider costs of HIV testing and counselling

	Ndirande clinic			Chilomoni clinic			QECH HTC clinic^a^			HIVST service		
Cost category	US Dollars (2014)	INT Dollars (2014)	% of Total^b^	US Dollars (2014)	INT Dollars (2014)	% of Total^b^	US Dollars (2014)	INT Dollars (2014)	% of Total^b^	US Dollars (2014)	INT Dollars (2014)	% of Total^b^
Staff salaries	6,738	24,545	17 . 9 %	6,433	15,019	11 . 1 %	8,710	24,195	12 . 5 %	23,066	79,431	30 . 3 %
Staff training	353	982	0 . 7 %	530	1,472	1 . 1 %	353	982	0 . 5 %	12,268	34,077	13 . 0 %
Monitoring and evaluation	2,098	5,828	4 . 3 %	5,785	16,069	11 . 9 %	2,920	8,111	4 . 2 %	15,833	54,521	20 . 8 %
Consumables and equipment	38,453	96,475	70 . 5 %	40,910	94,070	69 . 6 %	60,324	126,995	65 . 5 %	82,133	94,051	35 . 9 %
Capital/Overheads	3,257	9,047	6 . 6 %	3,102	8,618	6 . 4 %	12,129	33,691	17 . 4 %	0	0	0
Total annual health provider cost	50,899	136,876		56,760	135,248		84,436	193,973		133,300	262,080	
Individuals tested	6759			5372			9488			15190		
Direct cost per individual tested	7 . 53	20 . 25		10 . 57	25 . 18		8 . 90	20 . 44		8 . 78	17 . 25	
HIV positive identified	756			743			2984			1367^c^		
Direct cost per HIV positive identified	67 . 33	181 . 05		76 . 39	182 . 03		28 . 30	65 . 00		97 . 50	191 . 70	
Direct cost per HIV positive individuals assessed for ART eligibility	83 . 48	224 . 51		92 . 38	220 . 13		37 . 73	86 . 67		165.14	324.67	
										(173.05)^d^	(340.23)^d^	
										(233.90)^e^	(459.86)^e^	
Direct cost per HIV positive initiated onto ART	109 . 85	295 . 40		132 . 42	315 . 52		85 . 75	196 . 98		319.67	628.50	

^a^Outpatient HIV Testing and counselling clinic at Queen Elizabeth Central Hospital

^b^Percentages based on costs estimated in International Dollars

^c^Estimated from HIV prevalence reported in main trial

^d^High linkage rate (56.3 %) from main trial used to estimate cost per individual assessed for ART eligibility [15]

^e^Low linkage rate (41.7 %) from main trial used to estimate cost per individual assessed for ART eligibility [15]

At the three health facilities, staff salaries accounted for between 11.1 % and 17.9 % of the total International Dollar provider costs; the values for staff training varied between 0.5 % and 1.1 %, monitoring and evaluation between 4.2 % and 11.9 %, and consumables and equipment between 65.5 % and 70.5 %. In comparison, for the HIVST service staff salaries accounted for 30.3 %, staff training for 13.0 %, monitoring and evaluation for 20.8 %, and consumables and equipment for 35.9 % of the total International Dollar provider cost.

Table 3 shows the time inputs, and direct non-medical and indirect costs, associated with accessing either modality of HTC. Most individuals who self-tested did not incur any costs, need a family member or carer to accompany them, or take time off work. Approximately 26.6 % (124/466) of all facility testers reported taking time off work to get tested, and 27 % (126/466) needed a family member or carer to accompany them to the testing facility. In comparison to HIVST, facility-HTC participants incurred a mean additional direct non-medical cost of US$0.84 (bootstrap 95 % CI: US$0.73-US$0.95), whilst indirect costs were elevated by an average of $1.41 (bootstrap 95 % CI: US$0.84-US$1.98) with the testing process taking an additional 177.5 minutes (95 % CI: 165.8-187.2). The mean combined direct non-medical and indirect cost of facility-HTC was US$2.93 (bootstrap 95 % CI: US$1.90-US$3.96) higher than for HIVST.

**Table 3:** Direct non-medical and indirect costs and time inputs

	Intervention clusters		Control clusters	Mean differences (95 % CI)^c^	
	HIVST (n = 775)	Facility HTC (n = 253)	Facility HTC (n = 213)	HIVST V All Facility HTC	Intervention Facility HTC v Control Facility HTC
Patient direct non-medical costs					
2014 US Dollars (mean/SE)	0 (0, 0)^a^	0 . 90 (0 . 09)	0 . 78 (0 . 06)	-0 . 84 (-0 . 95, -0 . 73)	0 . 12 (-0 . 10, 0 . 33)
2014 INT Dollars (mean/SE)	0 (0, 0)^a^	2 . 49 (0 . 25)	2 . 17 (0 . 16)	-2 . 32 (-2 . 63, -2 . 01)	0 . 32 (-0 . 28, 0 . 92)
Time to get tested (mean/SE)^b^	30 . 2 (1 . 8)	215 . 2 (7 . 0)	196 . 5 (6 . 9)	-176 . 5 (-186 . 9, -166 . 1)	18 . 7 (-0 . 9, 38 . 3)
Patient time off work Yes	13 (1 . 7 %)	63 (24 . 9 %)	61 (28 . 6 %)	-	-
Indirect costs					
2014 US Dollars (mean/SE)	0 (0, 0)^a^	1 . 07 (0 . 24)	1 . 93 (0 . 56)	-1 . 41 (-1 . 96, -0 . 86)	-0 . 87 (-2 . 11, 0 . 38)
2014 INT Dollars (mean/SE)	0 (0, 0)^a^	2 . 97 (0 . 67)	5 . 37 (1 . 55)	-3 . 91 (-5 . 44, -2 . 38)	-2 . 41 (-5 . 87, 1 . 05)
Family or carer accompanied Yes	13 (1 . 7 %)	65 (25 . 7 %)	61 (28 . 6 %)	-	-
Family/carer direct non-medical costs					
2014 US Dollars (mean/SE)	0 (0, 0)^a^	0 . 24 (0 . 04)	0 . 26 (0 . 04)	-0 . 25 (-0 . 31, -0 . 19)	-0 . 02 (-0 . 13, 0 . 10)
2014 INT Dollars (mean/SE)	0 (0, 0)^a^	0 . 68 (0 . 11)	0 . 72 (0 . 11)	-0 . 70 (-0 . 86, -0 . 54)	-0 . 04 (-0 . 36, 0 . 27)
Family/carer time to accompany to test (mean/SE)^b^	0 (0, 0)^a^	54 . 3 (6 . 8)	51 . 8 (6 . 4)	-52 . 4 (-43 . 3, -61 . 5)	2 . 5 (-16 . 5, 21 . 5)
Family/carer loss of income					
2014 US Dollars (mean/SE)	0 (0, 0)^a^	0 . 03 (0 . 02)	1 . 29 (0 . 95)	-0 . 59 (-1 . 43, 0 . 25)	-1 . 25 (-3 . 16, 0 . 65)
2014 INT Dollars (mean/SE)	0 (0, 0)^a^	0 . 09 (0 . 05)	3 . 57 (2 . 65)	-1 . 64 (-3 . 97, 0 . 69)	-3 . 48 (-8 . 70, 1 . 72)
Total direct non-medical and indirect costs					
2014 US Dollars (mean/SE)	0 (0, 0)^a^	2 . 22 (0 . 27)	3 . 91 (1 . 09)	-2 . 93 (-3 . 94, -1 . 92)	-1 . 69 (-3 . 88, 0 . 51)
2014 INT Dollars (mean/SE)	0 (0, 0)^a^	6 . 18 (0 . 74)	10 . 87 (3 . 02)	-8 . 14 (-10 . 94, -5 . 35)	-4 . 69 (-10 . 73, 1 . 36)

*SE* standard error

^a^Median and IQR reported because of low numbers incurring costs/taking time off work

^b^Time measured in minutes and includes travel to and from testing site, waiting time and counselling and testing time

^c^Bootstrapped estimates of mean differences and 95 % CI

The mean societal cost per participant tested for facility-HTC was US$11.84 (95 % CI: US$10.81-12.86) compared to US$9.23 (95 % CI: US$9.14-US$9.32) for HIVST. In the multivariate analysis (Table 4), after adjusting for individual characteristics and HIV test result, the mean societal cost of HTC was US$2.38 (95 % CI: US$0.87-US$3.89) lower for HIVST than for facility-HTC.

**Table 4:** Multivariate Analysis exploring relationship between modality of HIV testing and total societal cost of testing^a^

		Total societal cost			
		Model 1 (n = 1240)		Model 2 (n = 1237)	
		2014 US Dollars	2014 INT Dollars	2014 US Dollars	2014 INT Dollars
		Coef (95 % CI)	Coef (95 % CI)	Coef (95 % CI)	Coef (95 % CI)
Exposure	Control clusters: Facility HTC	Ref	Ref	Ref	Ref
	Intervention clusters: Facility HTC	-1 · 45 (-3 · 62, 0 · 73)	-4 · 24 (-9 · 99, 1 · 52)	-0 · 98 (-2 · 59, 0 · 63)	-2 · 97 (-7 · 07, 1 · 13)
	Intervention clusters: HIVST	-3 · 01 (-5 · 14, -0 · 88)	-12 · 52 (-18 · 23, -6 · 82)	-2 · 38 (-3 · 89, -0 · 87)	-10 · 82 (-14 · 79, -6 · 87)
HIV Test Result	HIV negative	Ref	Ref	Ref	Ref
	HIV positive	1 · 19 (-0 · 04, 2 · 41)	2 · 76 (-0 · 29, 5 · 81)	1 · 11 (0 · 24, 1 · 99)	2 · 57 (0 · 41, 4 · 72)

Model 1: adjusted for exposure, HIV test result, age and sex

Model 2: additionally adjusted for marital status, educational attainment, income and wealth quintile

Missing data for HIV test result: 1; missing data for educational attainment: 1; missing data for socio-economic position: 2

^a^Findings from Generalized Linear Model with Poisson distribution and Identity link function

The HIV test result and HRQoL outcomes are summarized in Table 5. There was no significant difference between facility testers who resided in the intervention and control clusters with regards to the mean EQ-5D utility score, the mean VAS score, the descriptive components of the EQ-5D measure or their responses to the SAH measure. A significantly smaller proportion of HIVST participants who tested HIV negative reported problems in four of the five EQ-5D dimensions than compared to facility testers who tested HIV negative (p < 0.001 for all dimensions excluding “usual activities”).

**Table 5:** Health-related quality of life of HIV testers

			Intervention clusters		Control clusters	HIVST v	Intervention Facility HTC v Control Facility HTC
						All Facility HTC	
			HIVST (n = 775)	Facility HTC (n = 253)	Facility HTC (n = 213)	*p*-value	*p*-value
HIV test result (n/%)	HIV negative		670 (86 . 5 %)	146 (57 . 7 %)	115 (54 . 0 %)		0 . 421
	HIV positive		104 (13 . 4 %)	107 (42 . 3 %)	98 (46 . 0 %)	<0 . 001	
	Not reported		1 (0 . 1 %)	0 (0 %)	0 (0 %)		
EQ-5D: Utility score (mean/SE)	All		0 . 905 (0 . 897, 0 . 913)	0 . 828 (0 . 812, 0 . 844)	0 . 839 (0 . 821, 0 . 857)	<0 . 001	0 . 359
	HIV negative		0 . 916 (0 . 908, 0 . 924)	0 . 853 (0 . 834, 0 . 873)	0 . 862 (0 . 839, 0 . 884)	<0 . 001	0 . 591
	HIV positive		0 . 842 (0 . 814, 0 . 870)	0 . 794 (0 . 768, 0 . 819)	0 . 813 (0 . 786, 0 . 840)	0 . 022	0 . 306
EQ-5D: VAS score (mean/SE)	All		82 . 1 (81 . 0, 83 . 3)	74 . 5 (72 . 2, 76 . 8)	75 . 4 (72 . 9, 78 . 0)	<0 . 001	0 . 597
	HIV negative		83 . 7 (82 . 5, 84 . 9)	79 . 4 (76 . 6, 82 . 2)	79 . 5 (76 . 0, 82 . 9)	<0 . 001	0 . 966
	HIV positive		72 . 5 (69 . 0, 76 . 0)	67 . 9 (64 . 4, 71 . 3)	70 . 7 (67 . 0, 74 . 4)	0 . 135	0 . 270
EQ-5D: Mobility (n/%)	Moderate or severe problems	HIV negative	61 (9 . 1 %)	34 (23 . 3 %)	17 (14 . 8 %)	<0 . 001	0 . 085
		HIV positive	23 (22 . 3 %)	40 (37 . 0 %)	33 (34 . 0 %)	0 . 018	0 . 652
EQ-5D: Self-care (n/%)	Moderate or severe problems	HIV negative	5 (0 . 7 %)	2 (1 . 4 %)	1 (0 . 9 %)	0 . 551	0 . 707
		HIV positive	3 (2 . 9 %)	6 (5 . 6 %)	1 (1 . 0 %)	0 . 815	0 . 075
EQ-5D: Usual activities (n/%)	Moderate or severe problems	HIV negative	25 (3 . 7 %)	19 (13 . 0 %)	13 (11 . 3 %)	<0 . 001	0 . 676
		HIV positive	19 (18 . 4 %)	26 (24 . 1 %)	25 (25 . 8 %)	0 . 204	0 . 779
EQ-5D: Pain (n/%)	Moderate or severe problems	HIV negative	159 (23 . 8 %)	55 (37 . 7 %)	50 (43 . 5 %)	<0 . 001	0 . 342
		HIV positive	50 (48 . 5 %)	68 (63 . 0 %)	50 (51 . 5 %)	0 . 134	0 . 099
EQ-5D: Anxiety (n/%)	Moderate or severe problems	HIV negative	212 (31 . 7 %)	71 (48 . 6 %)	49 (42 . 6 %)	<0 . 001	0 . 333
		HIV positive	44 (42 . 7 %)	57 (52 . 8 %)	53 (54 . 6 %)	0 . 070	0 . 790
Self-assessed health (n/%)	Poor or very poor	HIV negative	6 (0 . 9 %)	6 (4 . 1 %)	5 (4 . 3 %)	0 . 001	0 . 924
	Poor or very poor	HIV positive	5 (4 . 9 %)	13 (12 . 0 %)	9 (9 . 3 %)	0 . 085	0 . 524

The mean EQ-5D utility score was higher amongst HIVST participants (0.905, 95 % CI: 0.897-0.913) than among facility testers residing in the intervention (0.828, 95 % CI: 0.812-0.844) or control (0.839, 95 % CI: 0.821-0.857) clusters. The mean VAS score for HIVST participants was also higher (82.1, 95 % CI: 81.0-83.3) than for facility-HTC participants residing in the intervention (74.5, 95 % CI: 72.2-76.8) or control (75.4, 95 % CI: 72.9-78.0) clusters.

In the multivariate analysis, the model diagnostics showed that the OLS estimator performed as well or better than the other estimators (Table 6). In the fully adjusted OLS model, the mean EQ-5D utility score was 0.046 (95 % CI: 0.022-0.070) higher in individuals who accessed HIVST than those who accessed facility-HTC. In those who tested HIV positive the adjusted mean EQ-5D utility score was 0.048 (95 % CI: 0.024-0.072) lower than in those who tested HIV negative. There were no significant differences in the adjusted mean EQ-5D utility scores between facility testers who resided in the control or intervention clusters.

**Table 6:** Multivariate analysis exploring relationship between modality of HIV testing and EQ-5D utility scores^a^

		EQ-5D Utility Score (Zimbabwean Tariff)		EQ-5D Utility Score (UK Tariff)^b^	
		Model 1 (n = 1240)	Model 2 (n = 1237)	Model 1 (n = 1240)	Model 2 (n = 1237)
		Coef (95 % CI)	Coef (95 % CI)	Coef (95 % CI)	Coef (95 % CI)
Mode of HIV testing	Control clusters: Facility HTC	Ref	Ref	Ref	Ref
	Intervention clusters: Facility HTC	-0 · 012 (-0 · 038, 0 · 014)	-0 · 011 (-0 · 037, 0 · 015)	-0 · 145 (-0 · 055, 0 · 026)	-0 · 012 (-0 · 053, 0 · 029)
	Intervention clusters: HIVST	0 · 043 (0 · 018, 0 · 068)	0 · 046 (0 · 022, 0 · 070)	0 · 059 (0 · 026, 0 · 092)	0 · 065 (0 · 031, 0 · 099)
HIV test result	HIV negative	Ref	Ref	Ref	Ref
	HIV positive	-0 · 054 (-0 · 077, -0 · 031)	-0 · 048 (-0 · 073, -0 · 024)	-0 · 076 (-0 · 112, -0 · 040)	-0 · 068 (-0 · 105, -0 · 031)

Model 1: adjusted for exposure, HIV test result, age and sex

Model 2: additionally adjusted for marital status, educational attainment, income and wealth quintile

Missing data for HIV test result: 1; missing data for educational attainment: 1; missing data for socio-economic position: 2

^a^Findings from OLS estimator

^b^Findings from sensitivity analysis

In the sensitivity analyses, when the UK tariff was used to derive EQ-5D utility scores, the adjusted mean EQ-5D utility score was 0.059 (95 % CI: 0.026-0.093) higher amongst HIVST participants than among facility testers (Table 6). In addition, those reporting a positive HIV test result had an even lower mean adjusted utility score compared to those who reported a negative HIV test result (mean decrement 0.068; 95 % CI: 0.031-0.105). Additional file 1 shows that the total societal cost of HIVST remained lower than for facility-HTC when alternative approaches to valuing loss of income were utilised.

## Discussion

In this study we found that, compared to facility-based HIV testing, HIVST reached a population who reported better health-related quality of life, with users incurring lower direct non-medical costs and work absences, whilst the direct health provider costs of offering HIVST were comparable to facility-based HTC. Consequently, from the societal perspective, the cost of providing HIVST was found to be significantly lower than facility-based HTC services. However, HIVST was costlier than facility-based HTC for identifying HIV positive individuals for treatment, as is typical for community-based services where HIV prevalence tends to be lower than in facilities [11, 12].

In the parent trial, uptake of HIVST was >70 % of all adult residents each year for two years [15], significantly greater than current use of facility-based HTC services in Africa [4]. However, the HIV prevalence amongst those accessing HIVST was lower, and fewer of those diagnosed HIV positive through HIVST linked into HIV treatment services than through facility-based HTC. Despite these limitations, well targeted community-based HTC services are considered essential to reaching UNAIDS 90-90-90 targets, due to low uptake of facility-based testing by men, adolescents, remote communities and key populations [4, 12]. In this context, our data support HIVST as a potentially affordable approach to providing community services, with high uptake [15] and provider costs (US$8.78 per HIVST episode in 2014 prices) similar to or lower than mobile or home-based HTC (US$7.77 to US$33.54 in 2012 prices) [11]. The higher health provider cost per HIV positive individual initiated onto ART through HIVST highlights the need to consider complementary low-cost interventions that increase linkage into HIV services after HIVST.

The relatively high current cost of the oral fluid RDT kits (USD$4, or US$4.80 including shipping and insurance), compared to US$0.69 for standard finger-prick RDT kits used in health facilities, explains much of the variation. In our analysis the cost of oral fluid RDT kits accounted for half of the total cost per individual tested through HIVST, whilst finger-prick RDT kits accounted for only one tenth of the cost of facility-based HIV testing. In 2010 alone, nearly 100 million HIV testing episodes were undertaken in Africa [37]. Given the steady increase in uptake of HTC since then [3], and projected needs to meet global targets [5], manufacturers need to be aware of the massive potential market for low cost, easily useable and disposal HIVST kits. In the meantime, scaling-up HIVST will require donor-provision of self-test kits, ideally with negotiation of lower prices through bulk procurement for low- and middle-income countries.

We compared both health provider and societal costs of HIVST and facility-based HTC. Health care costing studies and economic evaluations predominantly adopt a health provider perspective as the findings are used to inform how best to allocate finite health care resources. Taking into account the costs at the societal level informs us on the wider impact of healthcare interventions on the economy as a whole, and may explain reasons for sub-optimal uptake by the population served. Previous research highlights that high direct non-medical and indirect costs act as a deterrent to accessing facility-based HTC services [6, 38, 39]. In comparison to HIVST, we found facility testing was associated with a mean additional direct non-medical and indirect cost of US$2.93. In Malawi approximately three-quarters of the population live on less than $2 a day [40]. It is clear that the high client costs of accessing facility-based HTC are likely to act as a deterrent, and this may partly explain the high levels of uptake of HIVST seen in the main trial [15].

HIV testing and counselling has been provided at health facilities in Africa for over a decade. HIV counsellors at health facilities are experienced in providing HTC, and monitoring and evaluation systems have evolved. In contrast, HIVST is still in its infancy, with concerns remaining (albeit not supported by current evidence) around potential social harms [41]. Consequently, in the main trial, HIVST was provided through a semi-supervised semi-restricted community distribution model that incurred considerable training and supervision costs: salaries, staff training, and monitoring and evaluation accounted for approximately two-thirds of the cost of delivering HIVST, in comparison to less than one quarter for facility-based HTC. Less costly HIVST distribution models will almost certainly develop as experience accrues (e.g. counselling provided by telephone hotlines), even without the anticipated reduction in the unit cost of kits.

We used the average yield from the HIVST service over the two years in operation to estimate the health provider cost per HIV positive individual identified through HIVST, assuming individuals were offered annual HIVST. In the main trial, there was a ‘prevalence round’ effect, with HIV prevalence amongst self-testers found to be higher in the first year than in the second [15]. It is likely that the costs per HIV positive individual identified will continue to rise over the years of operation as the number of undiagnosed HIV infected individuals in the community falls.

The EQ-5D measure provides two assessments of HRQoL, the EQ-5D utility score and the VAS score. The VAS score reflects individuals’ self-assessment of their health status, whilst the EQ-5D utility score reflects a general population preference for the overall health state delineated across five dimensions with the added benefit that utility scores can inform cost-utility analyses. In our study we found HIV self-testers reported higher VAS scores than those who tested in facility services. HIV self-testers do not have their test result communicated to them by a HIV counsellor, and the higher VAS scores suggests this does not negatively impact on an individual’s HRQoL. Those who self-tested reported higher EQ-5D utility scores than those who accessed facility testing services, even after accounting for differences in HIV test result and socio-demographic characteristics. Previous work from the main trial found the median CD4 count amongst HIV self-testers who initiated ART to be higher than facility-based testers who initiated ART [15]. Moving HIV testing into the community potentially reaches a population whose HIV infection has not advanced sufficiently to result in their attendance at a health facility.

Also notable is that, despite the high uptake of HIVST in the main trial [15], intervention cluster residents continued to access facility-based HTC services, highlighting the complementary nature of the two models of provision. Facility HTC provides services that cannot be replaced in community, such as diagnostic HIV testing for management of illness (e.g. TB patients) and provider initiated testing for prevention of mother-to-child transmission [42]. In contrast, community-based services are intended to provide healthy individuals with the means of knowing their status on a regular basis, and providing early linkage into HIV treatment and prevention services [14, 15].

This is the first study we are aware of that has estimated the costs of providing HIVST in Africa. We explored in detail all costs associated with HIVST, from the health provider and societal perspectives, compared with facility-based HIV testing, as well as the HRQoL of users of both modalities. We undertook a range of sensitivity analyses to explore the impact of alternative approaches to estimating total societal costs and for valuing HRQoL. This is not, however, a full economic evaluation, and we, therefore, cannot comment on whether or not scaling-up HIVST is a cost-effective option for sub-Saharan Africa. A full economic evaluation would need to incorporate the costs of providing subsequent HIV treatment and the health outcomes of HIV positive individuals identified through the two modalities, among other considerations.

## Conclusions

HIV testing services in Africa are in urgent need of substantial scale-up. HIVST offers a potential option, and offering it reduces the economic burden on clients. However, enhanced HIVST strategies may be needed to target higher risk individuals or to increase linkage into HIV treatment services amongst those found to be HIV positive. The affordability of HIVST would substantially improve if the costs of HIV self-test kits were lower, or if HIVST could be provided safely and effectively through less restricted and supervised models. Further work is needed to explore the cost-effectiveness of HIVST.

## Additional file

Additional file 1:
**Appendix A.** Direct health provider costing methods. **Appendix B.** Sensitivity Analysis for multivariate regression of total societal cost (Model 2). (DOCX 48 kb)
