# Supplementary material for: Cost and quality of life analysis of HIV self-testing and facility-based HIV testing and counselling in Blantyre, Malawi
Source: BMC Med. 2016 Feb 19;14:34. doi: 10.1186/s12916-016-0577-7 (PMC4759936; doi:10.1186/s12916-016-0577-7)
Supplement: Additional file 1: — Appendix A. Direct health provider costing methods. Appendix B. Sensitivity Analysis for multivariate regression of total societal cost (Model 2). (DOCX 48 kb) [file 12916_2016_577_MOESM1_ESM.docx]

**Online Appendix**

**Maheswaran H, Petrou S et al. Cost and quality of life analysis of HIV self-testing and facility-based HIV testing and counselling in Blantyre, Malawi**

Appendix A: Direct health provider costing methods

Appendix B: Sensitivity Analysis for multivariate regression of total societal cost (Model 2)

**Appendix A: Direct health provider costing methods**

We undertook interviews with the relevant personnel at each of the HTC services to quantify the resources used to provide HIV testing and counselling (HTC). We then costed each resource use item to estimate the total cost of the relevant HTC service. We divided resources used in providing HIV testing into: (1) staff salaries; (2) staff training; (3) monitoring and evaluation; (4) consumables and equipment; and (4) capital/overheads.

Staff salaries included employer contributions and fringe benefits. For staff training we included all training provided to staff that related specifically to service provision. For facility-based HTC, we included the cost of HTC refresher training, but did not include the cost of the initial HIV counseling training course. For HIVST, we included the cost of the initial HIV counseling training course, as the community workers were not previously trained as HIV counselors. We also included the cost of all other training provided to the community counselors providing HIVST, but excluded all training for research related activities. The costs of all staff training was annuitized over their useful life with an annual discount rate of 3%, with the useful life based on how often the training would be repeated.

The costs of consumables and equipment’s were obtained from the Malawi Ministry of Health (MoH) price catalogue, which includes the cost of shipping for imported goods. For items not supplied by the MoH, we used the on-land costs obtained from local suppliers. For items bought internationally (e.g. HIV Self-Test kits) we used the international price, and included the cost of shipping and insurance. Equipment costs were annuitized over their useful life with an annual discount rate of 3%. As the majority of the equipment was office equipment, we assumed their useful life to be 3 years.

The cost of monitoring and evaluation (M+E) was estimated based on the activities undertaken locally and centrally. For facility-based HTC, we asked all staff working at the facilities time spent doing M+E activities or providing local supervision, and allocated this cost to M+E. In addition, we included the costs of M+E visits from the HIV teams at the Blantyre District Health Office and Malawi Ministry of Health. For the HIVST service we included all M+E activities undertaken centrally by staff working on the main trial, but excluded research-related M+E activities. The M+E costs were based on the proportion of total working hours spent by personnel at the sites of interest.

Overhead and capital costs included the costs of utilities, security and building maintenance. For the Ndirande and Chilomoni health facilities, we obtained these costs from the Blantyre District Health office that manage the clinic. The HTC clinic at Queen Elizabeth Central hospital (QECH) is managed by the hospital administration. As QECH provides both inpatient and outpatient care, we allocated all capital and overhead costs based on the ratio of clinical personnel working in the HTC clinic to the total number of clinical personnel working at the hospital, and only included costs relevant to providing the outpatient HTC service. The costs of buildings were estimated from rental costs for equivalent space. The HIVST service did not incur any capital or overhead costs as it is provided in the community counselors’ homes at no additional cost.

**Appendix B: Sensitivity Analysis for multivariate regression of total societal cost (Model 2)***

|  | | Total Societal Cost | | | | | |
| --- | --- | --- | --- | --- | --- | --- | --- |
|  |  | **Sensitivity Analysis A**  **(n=1237)** | | **Sensitivity Analysis B**  **(n=1237)** | | **Sensitivity Analysis C**  **(n=1237)** | |
|  |  | 2014 US Dollars  Coef (95% CI) | 2014 INT Dollars  Coef (95% CI) | 2014 US Dollars  Coef (95% CI) | 2014 INT Dollars  Coef (95% CI) | 2014 US Dollars  Coef (95% CI) | 2014 INT Dollars  Coef (95% CI) |
| Exposure | Control Clusters: Facility HTC | Ref | Ref | Ref | Ref | Ref | Ref |
|  | Intervention Clusters: Facility HTC | 0·19  (-0·76, 1·15) | 0·35  (-1·66, 2·35) | -0·32  (-1·66, 1·03) | -1·07  (-4·41, 2·27) | 0·36  (-0·47, 1·18) | 0·82  (-0·89, 2·53) |
|  | Intervention Clusters: HIVST | -1·12  (-2·02, 0·22) | -7·30  (-9·57, -5·04) | -1·88  (-3·13, -0·62) | -9·38  (-12·69, -6·07) | -1·73  (-2·56, -0·92) | -8·97  (-11·10, -6·84) |
| HIV Test Result | HIV Negative | Ref | Ref | Ref | Ref | Ref | Ref |
|  | HIV Positive | 0·77  (0·45, 1·08) | 1·70  (0·97, 2·43) | 1·30  (0·63, 1·97) | 3·09  (1·44, 4·74) | 0·77  (0·47, 1·08) | 1·75  (1·02, 2·49) |

*Findings from Generalized Linear Model with Poisson distribution and Identity link function

**Sensitivity Analysis A**: Using median wage rate and self-reported time of work to value income loss

**Sensitivity Analysis B:** Using self-reported income and total HIV testing time to value income loss

**Sensitivity Analysis C:** Using median wage rate and total HIV testing time to value income loss
